# The microstructural abnormalities of cingulum was related to patients with mild cognitive impairment: a diffusion kurtosis imaging study

**DOI:** 10.1007/s10072-022-06408-x

**Published:** 2022-09-28

**Authors:** Yueyang Liu, Dongtao Liu, Mingyong Liu, Kun Li, Qinglei Shi, Chenlong Wang, Zhenyu Pan, Lichun Zhou

**Affiliations:** 1 Department of Neurology, Civil Aviation General Hospital, Beijing, China; 2 Department of Neurology, Beijing Chaoyang Hospital, Capital Medical University, No. 5, Jingyuan Road, Beijing, China; 3 Department of Radiology, Beijing Chaoyang Hospital, Capital Medical University, Beijing, China; 4 MR Scientific Marketing, Diagnosis Imaging, Siemens Healthineers China, Beijing, China

**Keywords:** Mild cognitive impairment, Cerebral small vessel disease, Cingulum, Diffusion kurtosis imaging, Changes

## Abstract

**Objective:**

Our study aimed to investigate the correlations between microstructural changes of cingulum and patients with mild cognitive impairment (MCI) by diffusion kurtosis imaging (DKI) technique.

**Method:**

A total of 104 patients with cerebral small vessel diseases (cSVD) were retrospectively enrolled in this study. According to Montreal Cognitive Assessment Scale (MoCA) scores, these patients were divided into MCI group (*n* = 59) and non-MCI group (*n* = 45). The general clinical data was collected and analyzed. The regions of interests (ROIs) were selected for investigation in cingulum. The values of DKI parameters were measured in each ROI and compared between the two groups, the correlations between DKI parameters and MoCA scores were examined.

**Results:**

Compared to non-MCI group, MCI patients had more severe white matter hyperintensities (WMHs) (*P* = 0.038) and lower MoCA scores (*P* < 0.01). MCI patients showed significantly decreased fractional anisotropy (FA), axial kurtosis (AK), mean kurtosis (MK), radial kurtosis (RK), and kurtosis fractional anisotropy (KFA) in the left cingulum in the cingulated cortex (CgC) region (all *P* < 0.0125). In the left CgC region, FA, AK, MK, RK, and KFA were positively correlated with MoCA scores (*r* = 0.348, 0.409, 0.310, 0.441, 0.422, all *P* < 0.001). Meanwhile, FA, AK, MK, RK, and KFA were also positively correlated with MoCA scores (*r* = 0.338, 0.352, 0.289, 0.380, 0.370, all *P* < 0.001) in the right CgC region.

**Conclusion:**

DKI technique could be used to explore the microstructural changes of cingulum in MCI patients and DKI-derived parameters might be feasible to evaluate MCI patients.

## Introduction

Cerebral small vessel diseases (cSVD) is a disease with a high prevalence related to age; it is mainly including WMHs, recent small subcortical infarct, prominent perivascular spaces (PVS), cerebral microbleeds (CMBs), lacunes, and atrophy [1]. The prevalence of WMHs increased from 50 to 95% at the age of 45–80 years [2]. The prevalence of brain microbleeds is 24%; it gradually increases with age and reaches up to 38.8% in the patients over the age of 80 years [3]. The recent studies based on Chinese populations have shown that lacunar infarction accounts for 38–46% of ischemic stroke [4–6].

cSVD is thought to be one of the main causes of MCI. Growing evidence indicates that cerebrovascular pathology is the most important contributor to dementia. However, pure vascular dementia is relatively uncommon, accounting only 10% of all dementia cases, whereas multiple-etiology dementia with a vascular component, most often in combination with Alzheimer’s disease (AD), is more common and accounts for approximately 30 to 40% of all dementia cases [7, 8]. MCI is a common condition encountered by clinicians, the prevalence in persons 60 years and older was estimated between 15 and 20%, and the annual rate in which MCI progresses to dementia varies between 8 and 15% per year [9]. A meta-analysis that assessed the reversion rates from MCI to normal cognition in 25 studies indicated an overall reversion rate of approximately 24% [10]. The most cognitive impairment in the elderly arises from multiple pathologies, of which the vascular component is currently one of the treatable and preventable etiologies. Therefore, early recognition and timely intervention of MCI patients with cSVD may slow the progression to dementia. Several studies have confirmed that cSVD contributes to multiple domains of cognitive impairment [2, 11, 12]. This is thought to be the result of disruption of white matter (WM) tracts. The cingulum bundle is one of the most distinctive WM tracts, which interconnects frontal, parietal, and medial temporal sites, while it is also linking subcortical nuclei to the cingulate gyrus [13]. A recent study by Metoki found that the microstructural abnormality of cingulum is related to mnemonic function in cSVD [14]. This result was consistent with a number of previous studies of correlations between microstructural changes in the cingulum and MCI or AD [15–21]. However, all the studies mentioned above were analyzed through diffusion tensor imaging (DTI) methods, which cannot accurately describe the complexity of tissue microstructure because of the complexity of tissue structure and cell components. DKI can partially overcome these limitations and DKI parameters have been found to be very sensitive to identify certain changes in many neurological diseases [22, 23]. DKI can detect these microstructural changes even before any imaging findings through conventional imaging, that the reason why it is better than DTI [24]. This study aimed to investigate the correlation between the microstructural changes of cingulum and MCI patients with cSVD by DKI technology, which may provide neuroimaging evidence for the early evaluation of MCI patients.

## Material and method

### Subjects

We retrospectively collected 104 patients with cSVD from January 2018 to December 2019. The diagnosis was confirmed by conventional head magnetic resonance imaging (MRI) scan and magnetic resonance angiography (MRA) of the head [25]. All the participants underwent a baseline evaluation, including clinical data collection, cognitive function, and neuropsychological assessment. Inclusion criteria were as follows: age of ≥ 50 years, cranial MRI confirmed the presence of cSVD, mainly including lacunar infarcts and/or WMHs [1], complete cognitive and neuropsychological assessment, complete head MRI, including T1-weighted images (T1WI), T2-weighted images (T2WI), fluid-attenuated inversion recovery (FLAIR), diffusion-weighted imaging (DWI), susceptibility-weighted imaging (SWI), DKI, and MRA. Exclusion criteria were as follows: patients with severe neurological diseases, such as AD, Parkinson’s disease or brain trauma; patients with intracranial and external large vascular stenosis; patients with severe medical diseases, such as renal failure, liver diseases, heart diseases, or other systemic diseases; patients with severe mental disease or neuropsychological disorders; patients with low image data quality.

### General criteria for MCI

MCI diagnostic criteria were in compliance with the International Working Group on MCI [26]. Firstly, the participants should be judged neither normal nor dementia; Secondly, functional activities of the participants are mainly preserved, or at least that impairment is minimal; Thirdly, the participants should have evidence of cognitive decline, measured either by self and/or informant report in conjunction with deficits on objective cognitive tasks, and/or evidence of decline over time on objective neuropsychological tests.

### Cognitive function and neuropsychological assessment

All patients were evaluated by neuropsychological scales at admission. Mini-mental state examination (MMSE) and MoCA scales were applied to assess overall cognitive function and interpret the score according to their social/educational status. MoCA scores were ≤ 13 points for illiterate patients, ≤ 19 points for primary school patients, ≤ 24 points for junior high school and above patients, and the clinical dementia rating (CDR) ≤ 0.5 were considered to be MCI group, otherwise were considered to be non-MCI group [27]. We also assessed the severity of depressive or anxiety disorders by 24 stems of Hamilton Depression Scale (HAMD) and the Hamilton Anxiety Scale (HAMA). Patients with HAMD score < 7 points were considered normal; 7 to 17 points were considered mild depression; 18 to 24 points were considered moderate depression; and > 24 points were considered major depression. Patients with HAMA > 29 were divided into severe anxiety, 22 to 29 have markedly anxious, 15 to 21 have definitely anxious, 8 to 14 were likely to have anxiety, and < 8 was no anxiety symptoms.

### Assessment of WMHs

**Fig. 1:**
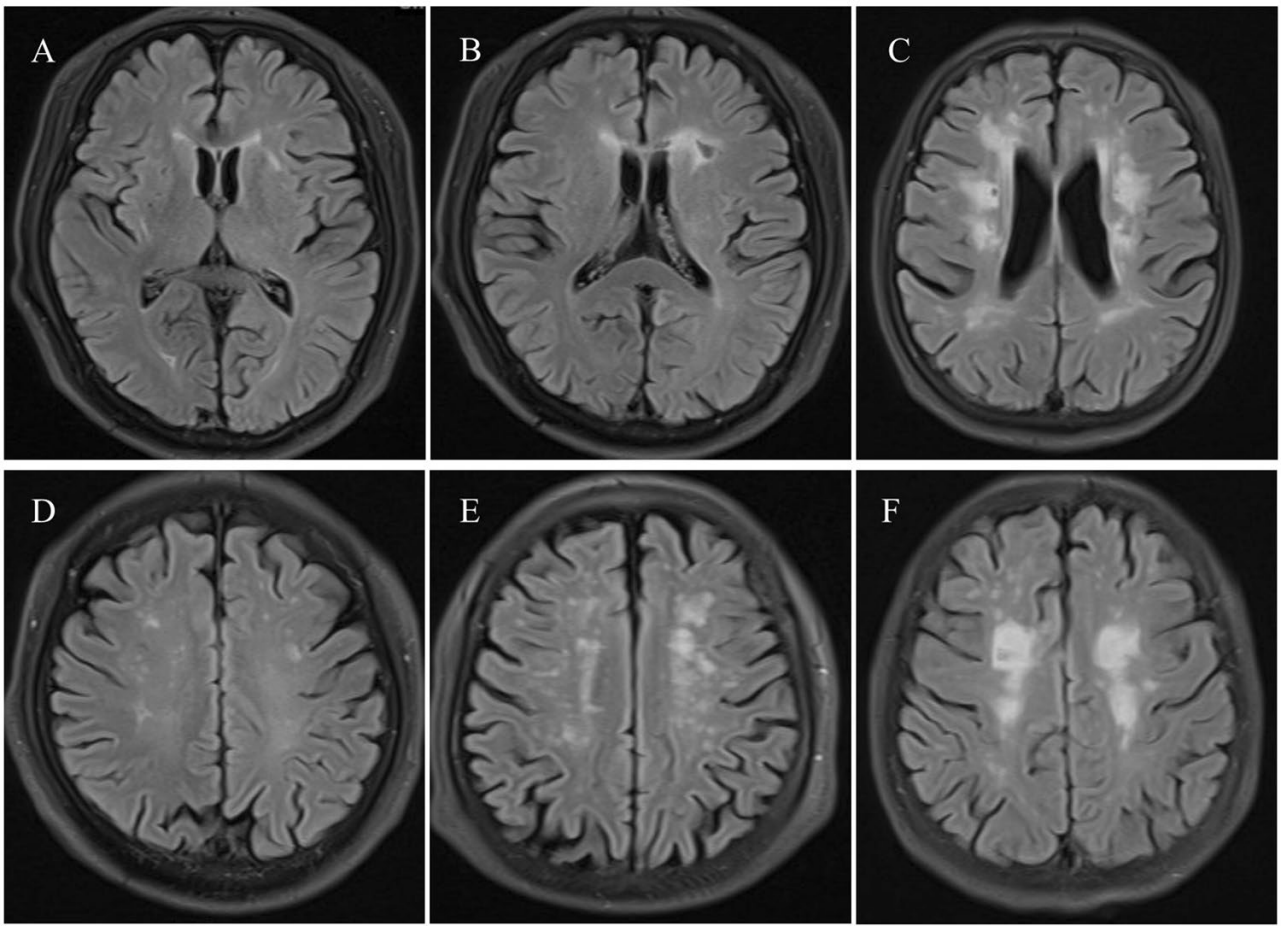
Differing severity of P-WMHs and D-WMHs. Magnetic resonance images showing severity in P-WMHs: (**A**) mild, (**B**) moderate and (**C**) severe. Magnetic resonance images showing severity in D-WMHs: (**D**) mild, (**E**) moderate and (**F**) severe

Assessments of WMHs were performed by two experienced radiologists (K. L. and Z. P.). Discrepancies were resolved by consensus. The *k* statistic of the intra-rater and inter-rater agreement was 0.85 or above, indicating good reliability. WMHs were defined as diffuse or confluent white matter hyperintensities in the periventricular or subcortical white matter observed on T2WI or FLAIR. WMHs were scored by using the Fazekas Scale. A detailed description of these assessments has been previously described. Periventricular white matter hyperintensities (P-WMHs) and deep white matter hyperintensities (D-WMHs) were evaluated separately and summed as Fazekas scores. The degree of WMHs were rated by Fazekas scores (mild: 0 to 2; moderate: 3 to 4; severe: 5 to 6; see Fig. 1) [28].

### MRI acquisition

Two radiologists viewed these images. All patients were scanned on a 3 Tesla scanner (MAGNETOM Skyra, Siemens Healthcare, Erlangen, Germany) with a 20-channel phased-array head coil. Foam fillers and earplugs were used to limit head movement and reduce scanner noise. T1WI were acquired using a 3D magnetization-prepared rapid acquisition gradient echo (MPRAGE). The parameters were set as follows: repetition time (*TR*) = 2300 ms, echo time (*TE*) = 89 ms, inversion time (*TI)* = 900 ms, flip angle (*FA*) = 8°, voxel size = 0.9-mm isotropic, parallel acceleration factor (*PAT*) = 2, field-of-view (*FOV*) = 240 × 240 mm^2^, and acquisition time = 5 min 21 s. Diffusion imaging was performed by using spin-echo plane imaging (SE-EPI) and scanned in two blocks. The sequence parameters of the first block were: *TR* = 7700 ms, imaging matrix = 74 × 74, *TE* = 89 ms, *FOV* = 222 × 222 mm, slice thickness = 3 mm, number of slices = 50, *b* = 0, 1000, 2000s/mm^2^, 30 gradient directions, 1 average, *PAT* = 2, and the acquisition time was 8 min 14 s. The parameters of the second block were the same as those of the first block, except that only *b* = 0 s/mm^2^ was used; the average was 9; and the acquisition time was 1 min 34 s. The total time of diffusion scan was 9 min 48 s.

### DKI data processing

**Fig. 2:**
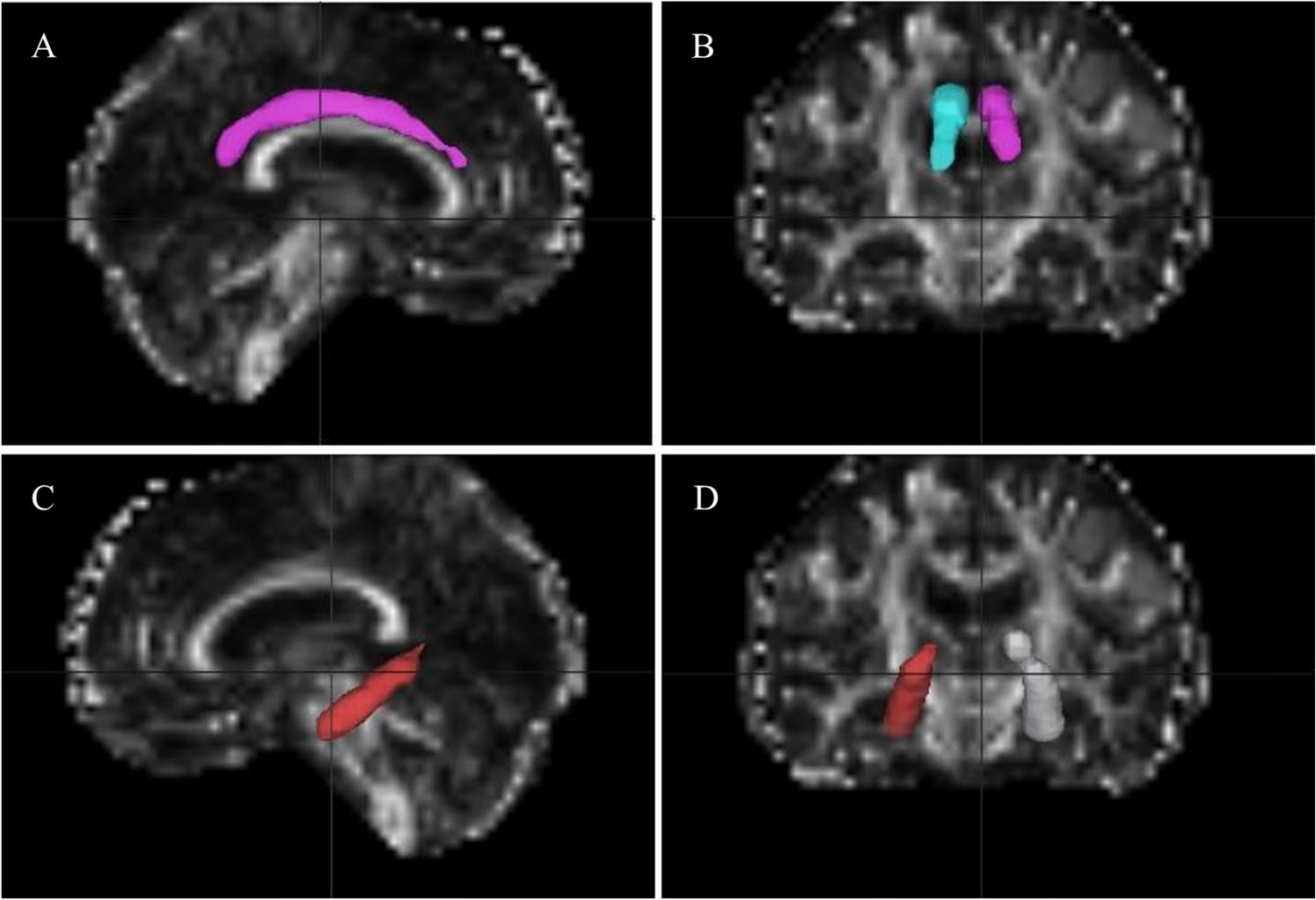
The CG in cingulate gyrus and hippocampal regions was separated at the axial level of the splenium of the corpus callosum and denoted as CgC (**A** and **B**), CgH (**C** and **D**). CgC: cingulum in cingulated cortex; CgH: cingulum in hippocampus

The diffusion images were first transformed to NII file format by using the dcm2nii tool, then, supplied to the diffusional kurtosis estimator (DKE) to generate DKI parameter maps. The T1WI acquired by MPRAGE were supplied to the SPM12 toolbox [29]. The DWI images (*b* = 0 s/mm^2^) were strictly aligned with T1WI space, and the transformed matrix was applied to the DKI parameter map. The DKI parameters of ROIs were automatically extracted by using MATLAB (2017a, The MathWorks, Inc., Natick, MA). The parameters of DKI include: MD, AD, RD, FA, MK, AK, RK, and KFA. MK, the most commonly used DKI parameter, means the average of the diffusion kurtosis along all diffusion directions; AK is the kurtosis along the axial direction of the diffusion ellipsoid; RK is the kurtosis along the radial direction of the diffusion ellipsoid; and FA is the most commonly used DTI parameter, which has been a primary imaging metric used in the evaluation of a wide range of neuropathologic processes [30]. The cingulum (CG) in the cingulate gyrus and the hippocampal regions is separated at the axial level of the splenium of the corpus callosum and denoted as CgC and CgH, respectively. CgC and CgH were selected as ROIs according to the ICBM template (see Fig. 2) [31].

### Statistical analysis

Statistical analyses were performed using SPSS (version 20.0, IBM Corp., Armonk, NY). The one-sample Kolmogorov–Smirnov test was used to test the normality of the data distribution. Data was expressed as mean ± standard deviation ($\overline{X }$ ± SD) or median (quartile). The Mann–Whitney *U* test, the independent *t-*test, or the χ^2^ test were applied appropriately for comparison between the two groups. The Bonferroni method was used to correct the *P* value, and the corrected *P* value was statistically significant when *P* < 0.0125 (0. 05/4 = 0.0125). Correlation between the MoCA scale scores and DKI parameters was evaluated with Spearman correlation analysis. A value of *P* < 0.05 was considered statistically significant.

## Results

### Comparison of general characteristics

According to the presence or absence of MCI, the 104 patients were divided into MCI group (59 cases) and non-MCI group (45 cases). In MCI group, there were 35 males, the ages ranged from 50 to 88 years old, with a median age of 65 (60, 72) years old, the education period was from 0 to 18 years, and the median duration of education was 8 (7, 10) years. In the non-MCI group, there were 26 males; the ages ranged from 50 to 78 years old, with a median age of 64 (58, 69) years old; the duration of education was from 8 to 11 years, and the median duration of education was 9.5 (8, 11) years. There were no significant differences in age, gender, and years of education between the two groups (*P* > 0.05).

Patients with MCI had more severe total WMHs (*P* = 0.038) and had evident decreased cognitive function scores. MMSE and MoCA scores were significantly different between the two groups (both *P* < 0.001). There were no significant differences in the risk factors of cerebrovascular diseases (such as diabetes mellitus, hypertension, and history of smoking) and the blood test results (such as serum glucose, total cholesterol, and serum homocysteine) between the two groups (see Table 1).

**Table 1:** General characteristics and cognition function of MCI and non-MCI patients

	MCI group *N* = 59	Non-MCI group *N* = 45	*U*/*χ*^2^	*P*
Age [year, M (P_25_, P_75_)]	65 (60,72)	64 (58,69)	− 0.765	0.329
Sex (male, *N*, %)	35 (59.3)	26 (57.8)	0.053	0.645
Education [year, M (P_25_, P_75_)]	8 (7,10)	9.5 (8,11)	− 1.708	0.088
Hyperlipidemia [*N*, %]	27 (56.3)	15 (44.1)	1.173	0.279
DM [*N*, %]	23 (39.1)	18 (40.2)	0.146	0.956
Hypertension [*N*, %]	40 (67.8)	28 (62.2)	0.442	0.531
History of drinking [*N*, %]	16 (27.1)	14 (31.1)	1.694	0.468
History of smoking [*N*, %]	22 (37.3)	18 (40.0)	0.142	0.894
Glucose[mmol/L, M (P_25_, P_75_)]	5.5 (4.8,6.6)	5.3 (4.6,7.2)	− 0.118	0.814
UA [umol/L, M (P_25_, P_75_)]	285 (230,392)	281 (252,355)	− 0.381	0.712
CR [umol/L, M (P_25_, P_75_)]	69.3 (62.9,85.1)	68.2 (57.1,76.2)	− 1.589	0.986
TC [mmol/L, M (P_25_, P_75_)]	4.5 (3.5,4.8)	4.4 (3.8,5.2)	− 0.634	0.563
TG [mmol/L, M (P_25_, P_75_)]	1.4 (1.0,1.8)	1.3 (0.8,1.9)	− 0.952	0.381
LDL-C [mmol/L, M (P_25_, P_75_)]	2.4 (1.8,2.7)	2.3 (2.0,2.7)	− 0.442	0.658
LPa [mmol/L, M (P_25_, P_75_)]	173 (72,386)	134 (53,181)	− 1.312	0.162
HCY [mmol/L, M (P_25_, P_75_)]	14.2 (12.1,16.4)	12.5 (10.7,15.2)	− 1.723	0.067
Glycated hemoglobin	6 (5.7,6.7)	6.3 (5.7,6.7)	− 0.968	0.329
Total-WMHs (Fazekas scale)			6.552	0.038
Mild (0–2)	11 (18.8%)	17 (38.2%)		
Moderate (3–4)	26 (43.8%)	21 (47.1%)		
Severe (5–6)	22 (37.5%)	7 (14.7)		
MoCA [scores, M (P_25_, P_75_)]	21 (18,22)	27 (25,28)	75.50	0.000
MMSE [scores, M (P_25_, P_75_)]	27 (25,28)	30 (29,30)	201.00	0.000

*DM* diabetes mellitus, *UA* uric acid, *Cr* creatinine, *TC* total cholesterol, *TG* triglyceride, *LDL-C* low density lipoprotein cholesterol, *Lpa* lipoprotein a, *HCY* homocysteine, *MoCA* Montreal Cognitive Assessment, *MCI* mild cognitive impairment, *WMHs* white matter hyperintensities, *MMSE* mini-mental state examination

### Neuropsychological test scores

The results showed that 27 of the 59 MCI patients had normal MMSE scores; the MoCA scale indicated that in addition to delayed recall impairment, MCI patients mainly combined with damage to visuospatial/executive, language, and abstract functions (see Table 2).

**Table 2:** Characteristics of MMSE and MoCA scores of patients in MCI group

Scales	MMSE		MoCA						
	≥ 27	< 27	Visuospatial/executive	Naming	Attention	Language	Abstraction	Delayed recall	Orientation
*N*	27	32	44	25	25	52	46	59	24
%	46%	54%	75%	42%	42%	88%	78%	100%	41%

*MMSE* Mini-mental state examination, *MoCA* Montreal Cognitive Assessment, *N* number

### Comparison of DKI parameters in cingulum between the MCI and non-MCI groups

Compared to non-MCI group, the MCI patients showed significantly increased MD and RD (*P* = 0.03, 0.02 respectively) and significantly decreased FA, AK, MK, RK, and KFA in the left CgC region (*P* = 0.002, 0.001, 0.001, 0.002, 0.005, respectively). No parameters were found to be significantly different between the two groups in the right CgC region, and both sides of the CgH regions. After correction by Šídák-Bonferroni method, FA, AK, MK, RK, and KFA still remained statistically different in the left CgC region (*P* = 0.002, 0.001, 0.001, 0.002, 0.005, respectively; see Tables 3–4).

**Table 3:** Comparison of DKI parameters in CgC in patients between MCI and non-MCI group

Group	MCI group N = 59		Non-MCI group N = 45		*t/U* value^a^	*P* value^a^	*t*/*U* value^b^	*P* value^b^
	Left	Right	Left	Right				
AD	1.45 ± .22	1.50 ± 0.24	1.38 ± 0.13	1.45 ± 0.17	1.22	.225	1.88	.064
MD	1.22 ± .20	1.27 ± 0.22	1.14 ± .12	1.21 ± 0.16	1.54	.126	2.21	.03
RD	1.10 ± .19	1.15 ± 0.21	1.02 ± .11	1.08 ± 0.15	1.72	.089	2.38	.02
FA	.17 ± .21	0.16 ± 0.03	.18 ± .02	0.17 ± 0.03	− 2.22	.029	− 3.17	.002
AK	.52 ± .04	0.52 ± 0.06	.56 ± .04	0.56 ± .06	− 2.45	.017	− 3.45	.001
MK	.60 ± .05	.59 ± .07	.64 ± .05	.62 ± .07	− 2.33	.023	− 3.50	.001
RK	.68 ± .07	.66 ± .09	.73 ± .06	.70 ± .08	− 2.20	.031	− 3.24	.002
KFA	.24 ± .03	.24 ± .04	.26 ± .03	.26 ± .04	− 1.98	.051	− 2.91	.005

*DKI* diffusion kurtosis imaging, *CgC* cingulum in cingulated cortex, *MCI* mild cognitive impairment, *AD* axial diffusion, *MD* mean diffusion, *RD* radial diffusion, *FA* fractional anisotropy, *AK* axial kurtosis, *MK* mean kurtosis, *RK* radial kurtosis, *KFA* kurtosis fractional anisotropy

^a^The right side group test value and *P* value

^b^The left side group test value and *P* value

**Table 4:** Comparison of DKI parameters in CgH in patients between MCI and non-MCI group

Group	MCI group *N* = 59		Non-MCI group *N* = 45		*t*/*U* value^a^	*P* value^a^	*t*/*U* value^b^	*P* value^b^
	Left	Right	Left	Right				
AD	1.14 (.75, 1.60)	1.17 (.75, 1.58)	.86 (.60, 1.36)	.98 (.68, 1.30)	639.00	.096	638.00	.094
MD	.91 (.62, 1.24)	.94 (.62, 1.27)	.66 (.49, 1.07)	.79 (.56, 1.08)	638.00	.094	633.00	.085
RD	.80 (.53, 1.07)	.82 (.54, 1.09)	.57 (.44, .91)	.70 (.50, .94)	639.00	.096	622.00	.068
FA	.15 (.09, .21)	.15 (.09, .21)	.12 (.08, .18)	.12 (.08, .16)	657.00	.135	690.00	.236
AK	.47 (.30, .67)	.52 (.34, .65)	.35 (.27, .56)	.37 (.31, .52)	634.00	.087	668.00	.164
MK	.51 (.32, .74)	.55 (.35, .72)	.39 (.28, .58)	.42 (.33, .56)	631.00	.082	672.00	.175
RK	.52 (.32, .74)	.58 (.37, .75)	.41 (.29, .58)	.43 (.34, .58)	630.50	.081	667.00	.161
KFA	.24 (.15, .37)	.24 (.16, .72)	.19 (.14, .31)	.20 (.14, .28)	662.00	.147	691.00	.239

*DKI* diffusion kurtosis imaging, *CgH* cingulum in hippocampus, *MCI* mild cognitive impairment, *AD* axial diffusion, *MD* mean diffusion, *RD* radial diffusion, *FA* fractional anisotropy, *AK* axial kurtosis, *MK* mean kurtosis, *RK* radial kurtosis, *KFA* kurtosis fractional anisotropy

^a^The right side group test value and *P* value

^b^The left side group test value and *P* value

### Correlations between DKI parameters and MoCA scale scores

In left CgC region, FA, AK, MK, RK, and KFA were positively correlated with MoCA scores (*r* = 0.348, 0.409, 0.310, 0.441, 0.422, all *P* < 0.001), and in the right CgC region, FA, AK, MK, RK, and KFA were also positively correlated with MoCA scores (*r* = 0.338, 0.352, 0.289, 0.380, 0.370, all *P* < 0.001). However, the AD, MD, RD of the left and right CgC regions had no correlations with MoCA scores, and the parameters in the CgH regions also had no correlations with MoCA scores (detailed Spearman coefficients are summarized in Table 5).

**Table 5:** Spearson's correlations with DKI parameters for MoCA scale scores

MoCA	Brain regions	AD	MD	RD	FA	AK	KFA	MK	RK
	CgC_R	− .085	− .131	− .152	.338*	.352*	.289*	.380*	.370*
	CgC_L	− .159	− .167	− .173	.348*	.409*	.310*	.441*	.422*
	CgH_R	− .100	− .113	− .117	− .036	− .092	− .063	− .088	− .079
	CgH_L	− .073	− .079	− .086	− .024	− .040	− .020	− .036	− .040

*CgC* cingulum in cingulate gyrus, *CgH* cingulum in hippocampal, *R* right, *L* left, *AD* axial diffusion, *MD* mean diffusion, *RD* radial diffusion, *FA* fractional anisotropy, *AK* axial kurtosis, *MK* mean kurtosis, *RK* radial kurtosis, *KFA* kurtosis fractional anisotropy

^*^P < 0.01

## Discussion

Our study found that compared to non-MCI group, MCI group had more severe WMHs patients. Our study also showed that MoCA scale was more sensitive than MMSE for MCI patients. In addition to delayed recall impairment, MCI patients mainly combined with damage to language, visuospatial/executive and abstract functions. Our study mainly found that MCI patients showed significantly decreased FA, AK, MK, RK, and KFA in the left CgC region. FA, AK, MK, RK, and KFA were significantly positively correlated with MoCA scores in both sides of the CgC regions, while the DKI parameters in the CgH regions had no significant correlations with MoCA scores.

MMSE and MoCA were two widely used cognitive function assessment scales in clinical practice. The meta-analysis found that the sensitivity of MoCA to MCI patients was 80.4%, and the specificity was 81.19%. However, the sensitivity and specificity of MMSE to MCI patients were 66.34% and 72.94%, respectively [32]. Approximately 46% of the MCI patients in our study had normal MMSE scores, suggesting that MMSE had poor sensitivity to MCI patients, which is consistent with the results of previous study [32]. Our study showed that all patients with MCI had delayed recall impairment, and most patients accompanied with multiple cognitive domain impairments. Our results were consistent with those found by Papma et al., which indicated MCI patients with cSVD showed a cognitive profile of prominent memory impairment, dysexecutive functioning, and language problems, when compared with controls [33]. Similar to our results, Boyle et al. also found WMH volume was associated with an increased rate of decline in global cognition, including perceptual speed, working memory, episodic memory, and semantic memory [34]. However, different from our results, previous studies have also shown that patients with vascular brain lesions were impairments mainly in executive function and processing speed [35, 36]. Delayed recall of words in a verbal learning test is a sensitive measure for the diagnosis of amnestic mild cognitive impairment (aMCI), and aMCI is the typical prodromal stage of dementia due to AD [9]. Iadecola et al. found that AD is the leading cause of clinically diagnosed dementia in Western countries; cognitive impairment of vascular etiology is the second most common cause and may be the predominant one in East Asia [35]. As most cognitive impairment in the elderly arises from multiple pathologies, the population enrolled by our study maybe mainly represented with mixed etiology cognitive impairment (mostly vascular + degenerative AD-type).

Our study mainly found that compared to non-MCI group, the MCI patients showed significantly decreased FA, AK, MK, RK, and KFA in the left CgC region. The cingulum was regarded as the core part of the limbic system and also an important part of the cholinergic pathway. However, both the limbic system and the cholinergic pathway are related to cognitive impairment [37, 38]. This tract carries information from the cingulate gyrus to the hippocampus. Our results were consistent with a recent study, which found that individual FA differences in the dorsal/anterior cingulum contribute independently to all executive functions by Bettcher et al. [39]. Kantarci et al. employed ROI approach to explore the contribution of anterior and posterior cingulum FA and MD to executive/attention, language, memory, and visuo-spatial function in a group of 220 cognitive health older adults. They also found FA differences in the anterior cingulum were related to differences in attention/execution and memory, while FA seems to contribute to all four cognitive domains in the post cingulum [40]. Another study used by DTI tractography reconstructed cingulum and found individual FA differences in the anterior and posterior cingulum portion which was correlated with executive function tasks [17]. The reason may be that the anterior cingulum mainly correlated with attention and executive functions, while the parahippocampal cingulum will be more closely linked to learning and episodic memory [13]. The white matter microstructural changes in cingulum may be the reason for the decrease of FA; however, most of the studies on the microstructural changes of cingulum were conducted through the DTI method, and DKI technology was rarely applied [14, 15, 41]. Our previous study found that compared to non-MCI group, MCI patients showed significantly decreased MK in the left hippocampus (*P* = 0.002) [42]. We also found that MCI patients showed significantly decreased FA, AK, MK, RK, and increased MD and RD in the cingulate gyrus region [43]. Our results showed that kurtosis parameters were suggested to be more sensitive than diffusivity parameters for detecting microstructural changes in the cingulum. The reason for decreased of FA, AK, MK, RK, and KFA may be due to the loss of neuron cell bodies, synapses, and dendrites; the extracellular spaces were increased, and further research is needed to confirm our hypothesis.

Our study also found FA, AK, MK, RK, and KFA were significantly positively correlated with MoCA scores in both sides of the CgC regions. Kantarci et al. also found FA of parahippocampal cingulum was correlated with the severity of AD [44]. Likewise, the relationship between disease severity and cingulum microstructure comes from a study which also found the correlations between AD patients’ MMSE scores and MD in the posterior cingulum [45]. Our previous study also found in the left hippocampal region, FA, RK,MK, and KFA were positively correlated with MoCA scores [42]. Based on above studies, although the specific neurobiological mechanism behind the changes in kurtosis parameters was still unclear, the microstructure changes of DKI parameters may be caused by cerebral atrophy, or may appear before cerebral atrophy. However, the kurtosis and the diffusion parameters complement each other, and DKI technology may provide a certain reference value for the early diagnosis of MCI patients.

In addition, our study also revealed bilateral asymmetry in the microstructural changes of the cingulum in MCI patients. Compared to non-MCI group, the microstructural changes in the left cingulum were more obvious than in the right in MCI patients. Asymmetry plays an essential role in the healthy human brain, and changes in standard asymmetry patterns often mean pathological changes in the brain. Several studies focused on the relationship between abnormalities in brain symmetry and changes in the cognitive abilities. The big majority of these studies were on AD patients and in this population, the left hemisphere has been demonstrated to be significantly more impaired than the right, indicating a faster left hemisphere degeneration in AD[46–48]. However, there are only few studies on vascular dementia. A recent study about the changes in gray matter asymmetry and their relationship with cognitive impairment in patients with subcortical ischemic vascular disease (SIVD) found that in the fusiform and parahippocampal gyruses, the SVCI group displayed a dramatic rightward asymmetry [49]. WM asymmetries of the human brain have been well documented using diffusion tensor imaging (DTI) and revealed that cingulum was leftward asymmetry in human brains [47, 50]; our results were consistent with these studies. Our previous study also revealed that the microstructural changes in the left hippocampus were more obvious than in the right in MCI patients [42].

## Limitations of the study

Our study also has several limitations. Firstly, we have no normal healthy control patients; both of our two groups were cSVD patients, which may have a certain impact on the results. Secondly, because the MRI scan of the head of the enrolled patients took nearly 1 h, and most AD patients could not tolerate the long-time MRI scan, our study excluded AD patients. However, it has been clearly demonstrated that cSVD contributes to the development of AD and accelerates its progression [35]. So, this may have a certain impact on the results. Thirdly, the sample size of our study is relatively small, which may have contributed to the significant group difference. Furthermore, we did not analyze the volume of the cingulum, and the relationship between the volume of cingulum and the kurtosis diffusion parameters. Finally, due to the small number of MCI patients in our study, we did not further analyze the subtypes of MCI. Although we found that DKI technology has certain imaging diagnostic value in the early diagnosis of MCI patients, further research is still needed.

## Conclusion

DKI technology could be applied to observe the microstructural changes of the cingulum in MCI patients with cSVD. Compared to non-MCI group, some DKI parameters of cingulum were significantly different in MCI patents. Furthermore, some DKI parameters showed heterogeneous patterns of correlations with the MoCA scores of MCI patients, which might provide insights into the imaging evaluation of MCI patients.

## Data Availability

The datasets generated for this study were available on request to the corresponding author.
